# Preparation and *In vitro* / *In vivo* characterization of spray dried microsphere formulation encapsulating 4-chlorocurcumin

**DOI:** 10.4103/0250-474X.70481

**Published:** 2010

**Authors:** P. K. Gogu, A. V. Jithan

**Affiliations:** Vaagdevi College of Pharmacy, Ramnagar, Hanamkonda - 506 001, India

**Keywords:** Curcumin analogs, cirrhosis, spray dried microspheres, parenteral SR formulation

## Abstract

The objective of the present study was to prepare and characterize *in vitro* and *in vivo* performance of a sustained release microsphere formulation of 4-chlorocurcumin, a novel curcumin analogue. A spray dried technique with ethylcellulose as the polymer was used in the preparation of these microspheres. Microspheres were characterized for drug content, particle size and shape, *in vitro* drug release and the drug-polymer interaction. To assess *in vivo* performance, both pharmacokinetics and hepatoprotective activity were investigated. Results were compared with an equivalent i.v. solution. The microspheres of 4-chlorocurcumin with ethylcellulose were successfully prepared using a spray-dried technique. These microspheres were able to sustain the release of the drug both *in vitro* as well as *in vivo*. Microspheres offered better pharmacokinetic and hepatoprotective properties to the drug compared to its solution form.

Cirrhosis, a common liver disease, is characterized by diffused disorganization of normal hepatic structure by regenerative nodules that are surrounded by fibrotic tissue[1]. The pathological change in cirrhosis generally involves the entire liver and if neglected there is an irreversible damage to the liver. Unfortunately, there is no proper drug in the market either to stop the progress of this disease or cure it. However, several molecules demonstrated promise[2,3]. Development of new treatments and new delivery systems useful in cirrhosis is the need of the hour. Curcumin is a molecule which previously demonstrated promise in the treatment of this disease. Curcumin is 1,7 bis(4-hydroxy-3-methoxyphenyl)-1,6-heptadiene-3,5-dione or diferuloyl methane (fig. 1). Systemic delivery of curcumin is a huge challenge. Data from several studies indicated that its poor solubility, very low GIT dissolution rate, low absorption, and its extensive systemic metabolism are the reasons for its delivery problems. Several formulation and drug discovery strategies have been developed to overcome these delivery issues[4–6]. Several curcumin analogues synthesized also exhibited similar properties to that of curcumin and their delivery is also a challenge. This present study utilizing a curcumin analogue, called 4-chlorocurcumin (fig. 1), is a similar attempt on these lines. In this study, a newer curcumin analogue was evaluated for its potential and promising use in the treatment of cirrhosis and associated liver disorders. This preclinical study developed a sustained release system for the delivery of 4-chlorocurcumin and assessed its hepatoprotective activity. A sustained release system can prolong the release of the drugs at the site of action and reduce the fluctuations in drug levels, resulting in the effective therapy[7]. It can also reduce the side-effects. The study is based on the premise that drug delivery systems can be conveniently used in preclinical drug discovery stages with added advantages[8]. The poor oral bioavailability problem with curcumin and associated compounds can be weeded out using a SR parenteral microsphere formulation prepared using polymeric material. The first objective of this study was to prepare microspheres containing 4-chlorocurcumin using a spray drying technique with ethylcellulose as the polymeric material. The second objective of this investigation was to evaluate the pharmacokinetics and hepatoprotective activity after intraperitoneal administration of the developed formulation containing 4-chlorocurcumin.

**Fig. 1:**
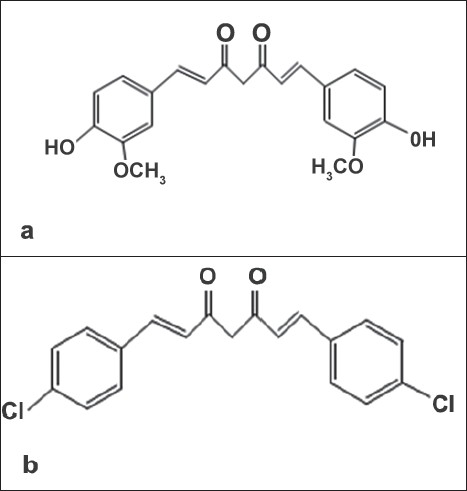
Structure of curcumin (a) and 4-chlorocurcumin (b)

## MATERIALS AND METHODS

Ethylcellulose was procured from Remedy Labs, Ahmedabad, India. All solvents used were procured from Finar Chemicals, New Delhi, India. All phenolic aldehydes used in the synthesis of 4-chlorocurcumin were procured from Sigma-Aldrich, New Delhi. Serum glutamic oxaloacetic transaminase (SGOT) and serum glutamic pyruvic transaminase (SGPT) kits were procured from Zeal Chemicals, Hanamkonda, India. Male Wistar rats (150-250 g), purchased from Mahaveera Enterprises, Hyderabad, were used in this investigation. The present study protocol was approved by the Institutional Animal Ethics Committee (IAEC) of Vaagdevi College of Pharmacy, Ramnagar, Hanamkonda, AP, India. Spray gun used in the manufacture of the microspheres was procured from VJ Instruments, Pune. Elico SL 164 Double Beam Spectrophotometer was used in the assay of the samples. A HPLC system from Cyberlabs, Millbury, USA was used in the assay of the compound in the plasma samples. Magnetic stirrers from Remi Industries, Mumbai, India, were used. Research binocular microscope from Unomed health care, Chennai was used for taking pictures of the microspheres and the histology slides.

### Preparation and *in vitro* characterization of 4-chlorocurcumin microspheres:

4-chlorocurcumin was synthesized using a modification of a previously reported method[9,10]. After synthesis, purity was further checked with a HPLC using methanol:water at a ratio of 70:30 as a mobile phase at a detection wavelength of 425 nm. To prepare 4-chlorocurcumin microspheres, the drug and the polymer (ethyl cellulose) were taken in different ratios and were dissolved in 1% w/v of chloroform with the help of a sonicator. Three different ratios of drug:polymer: 1:12 (microspheres 1), 1:6 (microspheres 2) and 1:3 (microspheres 3) were selected for formulation preparation. Drug content taken in all the batches was 0.5 g. The drug-polymer solution thus prepared was poured into a spray gun. This was sprayed against the non abrasive glass as the substrate with specific spray time (6–7 s). After each specific spray, 2–3 min was given so as to aid the particle drying, formation of the particles on the substrate and the evaporation of the solvent. The spray number was kept at 15 times after which microspheres were collected by scrapping and further sieved thru sieve number 85 and 100. This methodology of preparation of microspheres was previously validated in our laboratory using placebo ethylcellulose microspheres. Drug particles were prepared following very similar method used for the preparation of placebo particles. The morphology of the particles was immediately noted using a microscope. The prepared microspheres were further characterized for particle size, particle characteristics (shape factor, ferrets diameter), percentage yield, encapsulation efficiency, *in vitro* drug release studies and drug polymer interaction. To characterize drug polymer interaction a FTIR was used.

To determine particle size, calibration of the microscope was done prior to the analysis. Approximately a cross section of 50 particles was taken and subsequently different diameters of the particles were determined. From this data particle information was obtained. The Feret diameter, F, is defined as the perpendicular distance between parallel lines, tangent to the perimeter at opposite sides of a 2D object in a certain direction. The shape factor (Q) is defined as the mean shape of Na measured objects according to the following Eqn, $Q\;=\;(4\pi /\mathrm{Na)}\;\times \;\sum_{i=1}^{\mathrm{Na}}\;\mathrm{Ai}/\mathrm{Ui2}$ where A is the area and U is the perimeter length of the objects. This shape factor varies between 0 and 1. For a sphere the factor is 1 and for a cube the mean shape factor is 0.668.

After fabrication the percentage yield was calculated using the formula, weight of the microspheres obtained after spray drying×100/amount of the drug and the polymer in the volume sprayed. To determine encapsulation, 20 mg of the microspheres were dissolved in 5 ml of chloroform. The absorbance of this solution was measured. The amount of the drug in the microspheres was subsequently determined using a standard curve previously generated by spiking a known amount of the drug into a polymeric solution of chloroform. To conduct *in vitro* release studies, commercially available dialysis membrane was soaked in distilled water for 30 min at 45° and a diffusion cell was prepared using a glass tube. In the donor 1 ml of microsphere suspension (20 mg 4-chlorocurcumin microspheres of all the batches prepared) dispersed in phosphate buffer (2 ml) was placed while the receiver contained 50 ml of distilled water. The receiver medium was stirred continuously while the samples were withdrawn at specific time point with the addition of equivalent buffer after the sample was taken. The samples were analyzed using UV/Vis spectrophotometer at 425 nm. To study drug polymer interaction, the FTIR spectra of polymer, placebo microspheres, drug and drug microspheres, ethyl cellulose and pure drug were obtained. From this data the drug polymer interaction was determined.

### Pharmacokinetics of 4-chlorocurcumin release from the microspheres:

From the data of the release studies, a suitable microsphere formulation to be used further in the rat studies was selected. For *in vivo* characterization, *in vivo* drug release (pharmacokinetic study) and reduction in hepatoprotective activity in a CCl_4_-treated rat model were determined. Pharmacokinetics of the formulation was studied in male Wistar rats. After procurement of the rats, these were acclimatized for 10 days prior to the conduction of the experiment. Rats were injected one time with microsphere suspension containing 2.8 mg of active drug intraperitoneally. A 20 mg microsphere suspension was dispersed in 1 ml normal saline and was injected. Parallel to the microsphere administration, another dose of the drug in the form of solution was administered via i.v. route in another set of rats. In this case drug was administered at an equivalent dose of 2.8 mg. Blood was periodically collected from retroorbital sinus and assayed for the drug using a HPLC standard developed using a previously reported method[9,10]. The mobile phase consisted of methanol:water at a ratio of 70:30. The retention times and the peak areas were noted. Extraction efficiency for the selected system was determined. The following possible pharmacokinetic parameters for both iv solution administration as well as ip microsphere administration were determined using WinNonlin: K_a_, k_e_, V_d_, elimination t_1/2_, absorption t_1/2_, Tmax, Cmax. Further, using Wagner-Nelson method cumulative amount of drug released into the plasma was determined.

### Determination of hepatoprotective activity of the microsphere formulation:

Hepatoprotective activity was determined for both the microsphere formulation and i.v. solution administration of 4-chlorocurcumin following a protocol reported previously. Another set of rats were used in this study. Male rats weighing around 150-200 g was used in this study. For this experiment, animals were divided into 6 different groups. Group I were normal control. Group II was given carbontetrachloride regularly and it served as toxic control. Group II received CCl_4_ at a dose of 0.7 ml/kg by intraperitonial route on the 3rd, 6th and 9th day consisting of mixture of CCl_4_ and olive oil (25:75). Group III received the drug in the solution form by i.v. route. Group III received 4-chlorocurcumin dissolved in PEG 600 solution at a rationale of 1 mg/ml by intravenous route twice daily. Group IV received 4-chlorocurcumin microsphere formulation one time in the beginning of the experiment. Microspheres 2 was administered for this purpose. A 20 mg of formulation was dispersed in 1 ml normal saline and was administered intraperitoneally. Group V were injected with placebo microspheres and Group VI served as a control for PEG injected and was injected 1 ml of PEG solution twice a day. Serum samples were procured from the blood samples collected from common carotid artery on the final day of experimentation prior to sacrifice of the animal. The blood samples collected were subjected to centrifugation for 15 min at 3000 rpm and the serum separated was transferred into fresh empty microcentrifuge tube and was stored in deep freeze for pharmacodynamic evaluation. Hepatoprotective activity was quantified by the activity of SGOT, SGPT levels present in the serum samples and the method used for quantification was modified IFFC method. Subsequently, livers of the all rats in each group were further sent for histopathological studies and for this purpose at the end of the study the rats were sacrificed, the livers were separated carefully and were transferred to sterile containers having formalin solution. Liver sections were subsequently prepared using a previously published method[2].

### Statistics

Students t-test was used to evaluate the significance of differences between groups. *p*<0.05 was considered statistically significant.

## RESULTS AND DISCUSSION

The poor absorption and bioavailability of curcumin in humans probably limits its clinical efficacy[4–6]. The need for curcumin-like compounds with improved bioavailability characteristics has led to the chemical synthesis of a series of analogues, using curcumin as the lead structure. In this study, the curcumin analogue 4-chlorocurcumin was selected for synthesis and aimed for the development of a microsphere formulation. The objective was also to evaluate the hepatoprotective activity and further investigate the pharmacokinetics of microsphere formulation. Formulation is an essential step that transforms active compound into successful products. For chronic diseases such as cirrhosis, SR dosage forms are desirable. A one time administration of SR dosage form is better when compared to repeated oral administration. Additionally, the drug of this study, 4-chlorocurcumin is a curcumin derivative, which is speculated to have a poor bioavailability and low half-life making it an ideal candidate for parenteral SR formulation. The potential of the curcumin and their analogs in curing various ailments right from arthrosclerosis to cancer can only be rediscovered by applying the new delivery systems. Poor bioavailability is the main issue that is being resolved with the use of 4-chlorocurcumin microspheres. In this study a drug included in a formulation was used for assessment of pharmacological activity. This screening method has several advantages as reported previously. Further, such a methodology slowly builds up formulation development right from the stage of drug discovery. These formulations if properly designed can lead to the market formulations also.

**Fig. 2:**
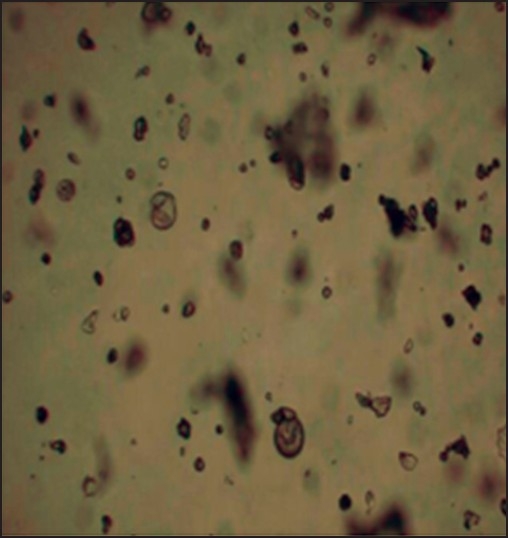
Photomicrograph of spray-dried 4-chlorocurcumin microspheres. Microspheres were viewed under an optical microspheres with a magnification of ×10

**Fig. 3:**
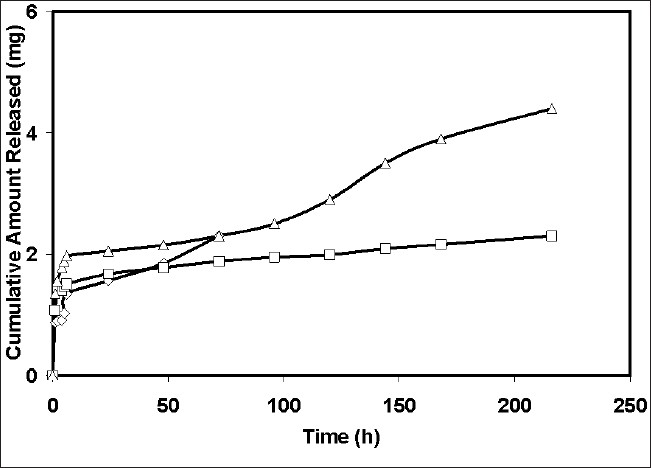
Drug release from different microsphere formulations. The release studies were performed in distilled water. The release data is from microspheres 1 (—◊—), microspheres 2 (—□—) and microspheres 3 (—∆—).

**Fig. 4:**
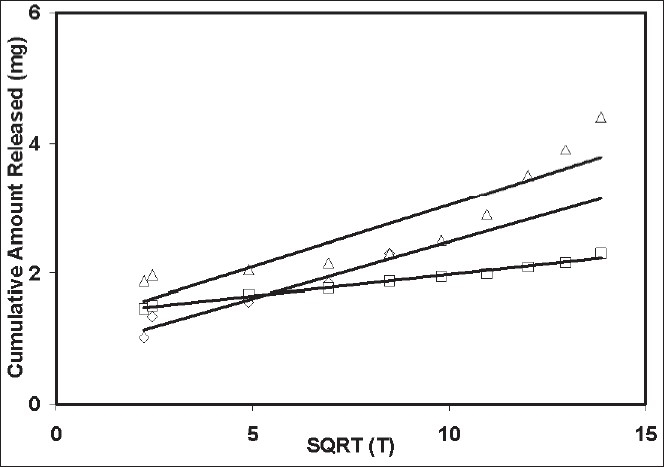
Higuchi plot for the release of 4-chlorocurcumin from microspheres. The release studies were performed in distilled water and the release data is shown in the fi gure, microspheres 1 (—◊—), microspheres 2 (—□—) and microspheres 3 (—∆—)

The compound of interest 4-chlorcurcumin was successfully prepared using the protocol mentioned in the methods[7,8]. This method is as modification of a previously published method. After synthesis, the purity was assessed using HPLC and it was found to be a pure compound containing only 0.3% starting material. The compound was also characterized by the peak purity assessment in HPLC. Subsequently, the formulations were prepared using the 4-chlorocurcumin synthesized. Microspheres with 4-chlorocurcumin encapsulated in a ethylcellulose matrix were successfully prepared using the spray-dried technique routinely being used in our laboratory. The pictures of the microspheres prepared using the spray dried technique developed in our laboratory is shown in fig. 2. Three different formulations microsphere 1, microsphere 2 and microsphere 3 were developed. The drug-polymer ratio in these formulations was 1:12, 1:6 and 1:3, respectively. At the end of fabrication, drug content was estimated in all the formulations. There was no loss of drug in the spray-dried process. Drug loading was 100%. However, the yield of the batches was 25%, 30% and 35%, respectively. Spray time and spray number of the method used was validated in our laboratory using placebo microparticles. Same parameters as those obtained for placebo particles were used for drug loaded particles. The morphology of the particles was noted immediately after the fabrication. The particles were spherical to approximately spherical. The ferrets diameters of the microsphere 1, microsphere 2 and microsphere 3 was 8.5, 11.73 and 15.5 mm, respectively. The shape factor was also determined and this was 0.912, 0.875 and 0.831 for microsphere 1, microsphere 2 and microsphere 3, respectively. This suggests that the particles although not spherical are closer to a sphere. *In vitro* release studies indicated that all the three formulations sustained the drug release (fig. 3). Microsphere 1 formulation sustained the drug release for 4 d with 100% release of the drug, while microsphere 2 formulation sustained the drug release for 8 d with 100% drug release and the microsphere 3 sustained the release of the drug for 16 d with 100% drug release. When Higuchi plot was drawn for the cumulative drug release, it was found that all the formulation had significant burst release for the first 3 days and then the drug release was sustained (fig. 4). With microsphere 3, although the release was significant, it did not follow a uniform pattern or Higuchi plot in the entire course of the drug release. However, the drug release from microsphere 2 followed a Higuchi release from day 3 to day 8, suggesting that the drug is releasing via a matrix diffusion process. Further, there was a uniform release. Since we were comparing the efficacy of the drug in ameliorating the hepatotoxity induced by carbon tetrachloride in the presence of dosage form and without dosage form, we wanted to have a formulation with consistent characteristics in terms of release. Further our model is a 9-day model and the microsphere 2 formulation sustains the release for 9 d. Thus, we used microsphere 2 formulation for further *in vivo* investigations. To see if there is a probable interaction between the drug and the polymer the samples of pure drug, 4-chlorcurcumin, microspheres, placebo microsphere formulation and pure ethyl cellulose were analyzed by the FTIR. FTIR study confers that there is no interaction between the polymer ethyl cellulose and 4-chlorocurcumin (data not shown).

**Fig. 5:**
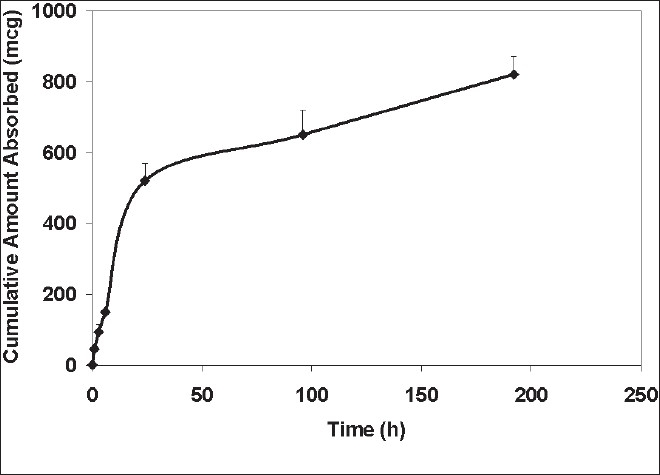
Drug release into the systemic circulation from the spray dried ethylcellulose microspheres. Cumulative amount was calculated using Wagner-Nelson method assuming the drug follows one compartment pharmacokinetics

In the animal experiments, various pharmacokinetic parameters with the drug delivery systems were calculated. Plasma drug levels were used in the calculation of the parameters. These levels were determined using a HPLC method developed. The mobile phase consisted of 70:30 methanol: water. Extraction efficiency of the method used was 80%. The retention time of the drug was 13 min. The UV/Vis detection wavelength was 425 nm and minimum detection level was 0.1 μg/ml. The PK parameters thus determined are shown in the Table 1. K_a_ and the absorption half-life calculated for the microsphere was 0.14/h and 5 h, respectively. Results indicated that, when microsphere 1 formulation was administered by IP routes, half-life (t_1/2_) and the volume of distribution (V_d_) were higher (*P*<0.05), and clearance (CL) was lower (*P*<0.05) than in the case of the free form injected by intravenous route (Table 1). When the formulation was administered by I.P. route, the area under the curve (AUC) and the time to peak concentration (t_max_) were significantly higher (*p*<0.05), and maximum concentration (C_max_) of 4-chlorocurcumin was lower than that of the free form. The cumulative amount of the drug absorbed as calculated using Wagner-Nelson method suggest that the drug released is sustained with an amount of 800 μg released into the systemic circulation during the 9 day of study period suggesting that the microsphere formulation was able to sustain the drug release after being injected intraperitoneally into the rats (fig. 5). The hepatoprotective activity of the formulation was also determined (Table 2). Hepatoprotective activity of microsphere 2 was clearly indicated from the decreased levels of SGOT and SGPT of the rats fed with the formulation administration in comparison with the toxic control CCl_4_. The PEG control as well as placebo microsphere control did not had any change in the SGOT and SGPT activity suggesting that these excipients are non toxic to the liver. Histopathological data also provided similar data. In the livers of rats treated with carbon tetrachloride, there was a significant necrosis of the liver cells including the parenchyma, whereas in all the other groups the histology revealed a normal liver. However, the percentage reversal with the i.v. solution administration both with SGOT and SGPT although statistically significant, was not reduced to reach the normal levels. However, histological studies revealed normal liver with both the formulations. Body weights of the rats were different with hepatotoxicity which was reversed with all the treatments suggesting body weight normalization with 4-chlorocurcumin. Thus, in this study a new molecule with a new delivery system for use in the treatment of cirrhosis and associated liver disorders has been developed.

**TABLE 1:** PHARMACOKINETIC PARAMETERS

PK parameters Calculated	IV 4-chlorocurcumin (2.8 mg)	IP microsphere (20 mg)
T_1/2_ (h)	1.987	82
V_d_ (ml)	23.52	50
CL (ml/h)	8.20	4.21
AUC (μg.h/ml)	227	300
T_max_ (h)	0.5	1.0
C_max_ (μg/ml)	196.43	39.82

**TABLE 2:** HEPATOPROTECTIVE ACTIVITY OF MICROSPHERE 2 FORMULATION

Groups	Body weights on day 9 (g)	SGOT (u/1)	SGPT (u/1)	Histo1ogy
Control	202±15	18.66±4.2	8.85±3.2	Normal liver
CCl_4_ treated	150±12	53.60±6.8	37.13±2.4	Massive necrosis and hepatitis of liver parenchyma
CCl_4_ treated+IV 4-chlorocurcumin solution	190±18	42.10±1.5	25.7±1.8	Normal liver
CCl_4_ treated+microsphere 2	185±15	32.94±3.8	18±1.5	Normal liver
Control+PEG solution	208±8	18.23±2.5	9.2±2.5	Normal liver
Control+Placebo microspheres	195±12	20.4±4.5	10.4±2.5	Normal liver
